# Safety and efficacy of combination therapy with low-dose gemcitabine, paclitaxel, and sorafenib in patients with cisplatin-resistant urothelial cancer

**DOI:** 10.1007/s12032-015-0683-y

**Published:** 2015-08-27

**Authors:** Yasuyoshi Miyata, Akihiro Asai, Kensuke Mitsunari, Tomohiro Matsuo, Kojiro Ohba, Hideki Sakai

**Affiliations:** Department of Urology, Nagasaki University Graduate School of Biomedical Sciences, Nagasaki, Japan; Department of Nephro-Urology, Nagasaki University Graduate School of Biomedical Sciences, 1-7-1 Sakamoto, Nagasaki, 852-8501 Japan

**Keywords:** Urothelial cancer, Paclitaxel, Gemcitabine, Sorafenib, QoL, Pain relief

## Abstract

Various regimens including molecular targeted agents have been examined in patients with cisplatin (CDDP)-resistant urothelial cancer (UC). However, some studies have been stopped owing to the development of severe adverse events. The main aim of this study was to examine the anticancer effects, changes in the quality of life (QoL), and safety of combined therapy of low-dose gemcitabine, paclitaxel, and sorafenib (LD-GPS) in patients with CDDP-resistant UC. Twenty patients were treated with gemcitabine (700 mg/m^2^ on day 1), paclitaxel (70 mg/m^2^ on day 1), and sorafenib (400 mg/day on days 8–22). QoL and pain relief were evaluated using the short-form survey (SF)-36 for bodily pain and the visual analog scale (VAS). VAS scores were significantly decreased by both the second- and third-line therapies (*P* = 0.012 and 0.028, respectively). The bodily pain score from the SF-36 survey was also significantly (*P* = 0.012) decreased. Complete responses, partial responses, and stable disease were found in 0 (0.0 %), 1 (5.0 %), and 13 patients (65 %), respectively. The median (interquartile range) period of overall survival after starting of this therapy was 7 (5–11) months. Three patients (15.0 %) stopped therapy because of grade 3 fatigue and hand–foot reactions. LD-GPS therapy was well tolerated by patients with CDDP-resistant UC. QoL was maintained, and improvements in their pain levels were found after treatment; pain relief was detected after third-line therapy. We suggest that this treatment regimen is worthy of consideration as second- and third-line therapy for patients with CDDP-resistant UC.

## Introduction

Urothelial cancer (UC) has a high prevalence rate among the elderly, and UC of the urinary bladder is the seventh most common cancer worldwide [1]. Bladder cancer patients with low-grade and low-stage disease are expected to be cured by local therapy, such as transurethral resection. In patients with early stage UC of the UT (UTUC), radical surgery should achieve a complete resection. On the other hand, outcome and survival of patients with metastatic and/or recurrent UC is poor. [2–4]. Over the last 20 years, chemotherapies using a cisplatin (CDDP)-based regimen have been considered the standard treatment in patients with advanced UC. Presently, combined chemotherapies, such as gemcitabine (GEM) and CDDP (GC regimen) and methotrexate, vinblastine, doxorubicin, and CDDP (MVAC regimen) are known to be the most effective regimens. However, only a small percentage of patients are cured completely with these CDDP-based combined regimens. In fact, the response rates of these chemotherapies are 40–50 %, and their median survival period is 14 months [5]. Unfortunately, there is no standard regimen for second-line therapy. Therefore, many investigators paid special attention to molecular and biological characteristics of UC cells. As a result, it is well known that advanced UC commonly has a higher frequency of changing of cancer-related molecules compared with non-muscle-invasive disease. Conversely, the cancer cells of advanced UC have many target molecules to suppress their malignant behavior. Therefore, various molecular-targeting agents have been used for clinical trials for patients with advanced UC [6]. However, almost all of the previous clinical trials that have used molecular-targeting agents have resulted in unsatisfactory efficacy and safety.

In recent years, a combination regimen of low dose of GEM and paclitaxel (PTX) has been reported to decrease the pain in patients with CDDP-resistant UC [7]. However, its anti-carcinogenic effects, including the decreasing of tumor mass volumes and prolongation of survival, are not satisfactory. On the other hand, this regimen has been shown to be safe and well tolerated by UC patients treated with CDDP-based chemotherapy. We hypothesized that a modified regimen based on LD-GP therapy plus a molecular-targeting agent may be effective and safe in patients with CDDP-resistant UC. Sorafenib is an oral multi-kinase inhibitor that inhibits malignant behaviors, including cell proliferation and angiogenesis [8–10]. In the present, sorafenib is commonly used worldwide for the management of several cancers, including renal cell carcinoma and hepatocellular carcinoma, because it has higher anticancer effects and be well tolerated in patients [11, 12].

This is the first report of a clinical trial of combination therapy with low dose of GEM, PTX, and sorafenib (LD-GPS) for patients with CDDP-resistant UC. The main aims of this study were to assess the following three factors: (1) safety and adverse events (2) maintenance of quality of life (QOL) after this therapy, and (3) anticancer effects including the changing of tumor size, survival, and pain relief. Finally, we demonstrate the usefulness and limitations of combination therapy of LD-GP and sorafenib as second-line and third-line therapy in patients with advanced UC.

## Patients and methods

Between June 2013 and August 2015, a total of 20 patients from Nagasaki University Hospital were enrolled into the study. An adequate bone marrow reserve was mandated for recruitment, with white blood cells >3000 mm^−3^, neutrophils >1500 mm^−3^, hemoglobin >8.0 g/dl, and a platelet count >100,000 mm^−3^ at entry. In addition, adequate renal and hepatic functions were required, with serum creatinine <2.0 mg/dl, total bilirubin <1.5 mg/dl, and aspartate aminotransferase <3 times the upper limit of normal. Prior to study entry, the Eastern Cooperative Oncology Group performance status was examined. In this study, all patients were treated with CDDP-based chemotherapy as first-line chemotherapy. The main exclusion criteria were uncontrolled hypertension and diabetes mellitus, New York Heart Association cardiovascular disease grades III–IV, respiratory failure, active infection, and incurable malignancies. In addition, patients with pure adenocarcinoma or squamous cell carcinoma without transitional cell carcinoma were also excluded. This study was carried out in accordance with the Declaration of Helsinki and Good Clinical Practice guidelines. This clinical trial was performed in a single hospital, and the study design and protocol were approved by the Ethics Committee (IRB) and Protocol Committee of Nagasaki University Hospital. All patients provided written informed consent.

PTX was given at 70 mg/m^2^ for 3 h by intravenous infusion on day 1. Similarly, GEM was administered at a dose of 700 mg/m^2^ intravenously for 30 min on day 1. Dexamethasone sodium phosphate (6.6 mg), diphenhydramine hydrochloride (50 mg), and ranitidine hydrochloride (100 mg) were administered before the administration of PTX. In addition, sorafenib was administered orally at a dose of 400 mg once daily on days 8–22. This schedule of combination chemotherapy with sorafenib was recycled every 28 days. The continuation of single-agent therapy with sorafenib or LD-GP was not permitted. During treatment, if an objective response or stable disease were obtained, the therapy was continued. When the patient or their family requested the cessation of the therapy, it was stopped immediately. Toxicities were graded according to the National Cancer Institute Common Terminology Criteria for Adverse Events, version 3.0. Similar to other previous studies, sorafenib was withheld until toxicity decreased to at least grade 2 in case of grade 4 myelosuppression and grade 3 toxicity [13].

All patients underwent a computed tomography (CT) scan and/or magnetic resonance imaging (MRI) to determine the in-field tumor response. The local response was assessed using the Response Evaluation Criteria in Solid Tumors guideline version 1.1. [14]. Based on these guidelines, complete response (CR) was defined as the disappearance of all target lesions and reduction of any pathological lymph nodes to <10 mm in the short axis. Partial response (PR) was defined as a decrease in the sum of the longest tumor diameters by at least 30 %. Stable disease (SD) was defined as neither sufficient shrinkage to qualify as PR nor sufficient increase in size to qualify as progressive disease (PD), which was defined as an increase in the sum of the longest tumor diameters by at least 20 %. In addition to the relative increase of 20 %, the sum had to also demonstrate an absolute increase of at least 5 mm. The appearance of new lesion(s) was also considered disease progression.

QoL was evaluated using the short-form questionnaire (SF-36), which comprises 36 questions measuring eight health scales. All SF-36 scores were linearly transformed to a scale of 0–100, where high scores reflect a better QoL. Pain was rated on a visual analog scale (VAS) of 0–10 (0 indicating no pain and 10 being the most severe pain imaginable). Data on QoL and pain were collected on the day before the first cycle was started and 8 weeks after starting the LD-GP plus sorafenib therapy. Positive pain relief was defined as a decrease in analgesic consumption or a decrease in VAS scores and bodily pain scores of the SF-36 without increasing the dose of analgesics. Regulation of the analgesic dose, including nonsteroidal anti-inflammatory drugs and opioids, was performed by an independent team who were unaware of the study.

Overall survival (OS) was measured from the first day of LD-GPS therapy to the day of patient death or last patient contact. Survival was plotted and analyzed using Kaplan–Meier curves and the log-rank *P* test. QoL score and changes in pain and analgesic consumption were evaluated in 17 patients who received at least two cycles of LD-GPS therapy. Data are expressed as the median and interquartile range (IQR). The Mann–Whitney *U* test was used for the analysis of continuous variables. The Chi-square test and Fisher’s exact test were used for categorical comparison of the data. All statistical tests were two-sided, and significance was defined as *P* < 0.05. All statistical analyses were performed on a personal computer with the statistical package StatView for Windows (version 5.0, Abacus Concept, Inc., Berkeley, CA).

## Results

As shown in Table 1, the median (IQR) age at the start of LD-GPS therapy was 74 (63–79) years. Eleven patients (55.0 %) were treated with chemotherapy and surgery, including transurethral resection. In regard to chemotherapy, all patients received a CDDP-based regimen including GC (*n* = 17, 85.0 %) as 1st-line chemotherapy. A total of 124 cycles were administered, and the median (IQR) number of treatment cycles per patient was 5 (3–8) cycles. Among 20 patients, 17 patients (85.0 %) received at least two cycles of chemotherapy and more than 12 cycles were given to three patients (15 %). To date, the maximum number of cycles is 20.

**Table 1:** Patient characteristics and previous therapies

Gender, *N* (%)	
Male	13 (65.0)
Female	7 (35.0)
Age at start of therapy, age	
Median (interquartile range)	74 (63–79)
Performance status, *N* (%)	
0	6 (30.0)
1	13 (65.5)
2	1 (5.0)
Primary site, *N* (%)	
Bladder/upper tract	10 (50.0)
Upper urinary tract	10 (50.0)
Sites of tumor, *N* (%)	
Lymph node	19 (95.0)
Primary lesion	9 (45.0)
Lung	8 (40.0)
Soft tissue	4 (20.0)
Bone	3 (15.0)
Others	5 (25.0)
Previous treatment, *N* (%)	
Chemotherapy and operation	11 (55.0)
Chemotherapy	7 (35.0)
Chemotherapy and radiation	2 (10.0)
Performed as second-line or third-line, *N* (%)	
Second	12 (60.0)
Third	8 (40.0)
Priory chemotherapy, *N* (%)	
First-line	
Gemcitabine plus cisplatin	17 (85.0)
Other cisplatin-based regimen	3 (15.0)
Second-line (*N* = 8)	
Gemcitabine and paclitaxel	4 (50.0)
Others	4 (50.0)

**Fig. 1:**
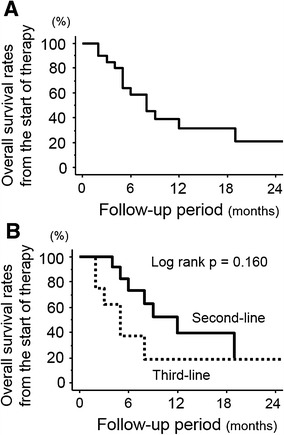
Kaplan–Meier survival curves of overall survival after starting low-dose gemcitabine, paclitaxel (LD-GP) plus sorafenib therapy. The median survival period was 7 months (**a**), which tended to be longer in the second-line setting compared with that observed in the third-line setting (**b**). However, this difference did not reach statistical significance

There were no patients with a CR. One (5.0 %) and 13 patients (65.0 %) showed PR and SD, respectively. On the other hand, six patients (30.0 %) were determined to have PD. In survival analyses, the median (IQR) period of OS after starting this therapy was 7 (5–11) months. Survival rates at 6 and 12 months after starting the therapy were 58.7 and 31.3 %, respectively (Fig. 1a). Similar survival analyses were performed to distinguish between second-line and third-line chemotherapies, and median/IQR periods with second-line therapy (median = 9/IQR = 6–14 months) had a trend toward being longer than those with third-line chemotherapy (3/5–7 months). Survival rates at 6 and 12 months in patients who received this regimen as second-line chemotherapy (73.3 and 39.3 %) were higher than that in those receiving it as third-line therapy (37.5 and 18.9 %). However, Kaplan–Meier survival curves showed no significant difference between these patient populations (*P* = 0.160, Fig. 1b).

**Fig. 2:**
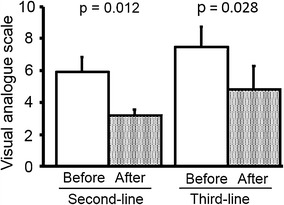
Scores on the visual analog scale of pain were significantly decreased by the therapy; such pain relief was found in both second- and third-line settings

**Fig. 3:**
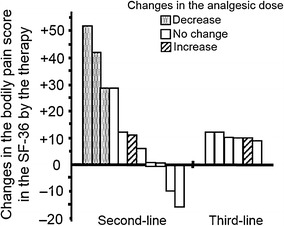
Changes in the bodily pain score in the short-form questionnaire (SF-36). In a second-line setting, remarkable pain relief was found in three patients. On the other hand, with the exception of one patient, bodily pain scores were also improved in the third-line setting

As shown in Table 2, VAS scores after the therapy were significantly (*P* = 0.001) lower than those before therapy. In second-line therapy, median/IQR of VAS scores after therapy (2/2–3) significantly decreased (*P* = 0.012) compared with their pretreatment levels (3/2–5). Likewise, a significant decrease was also found in third-line therapy (5/4–6 to 3/2–3, *P* = 0.028). Similar data for changes in VAS expressed as the mean and SD in second-line and third-line therapies are shown in Fig. 2. A decrease in analgesic consumption was seen in three patients (17.6 %), and two patients (11.8 %) had to increase the dose of analgesic (Fig. 3). Changes in SF-36 scores are shown in Fig. 3. Similar to the VAS scale, the bodily pain score was significantly (*P* = 0.012) decreased by the therapy. In addition, interestingly, increased bodily pain scores in the third-line setting were lower than those in the second-line setting; however, five of six patients had pain relief without increasing their analgesic consumption (Fig. 3). Role-physical scores tended to increase with therapy; however, this difference was not statistically significant (*P* = 0.103). On the other hand, the scores for other factors in pre- and post-treatment evaluations were similar. Finally, according to data for VAS, SF-36, and analgesic consumption, 11 of 17 patients (64.7 %) were determined to have pain relief.

**Table 2:** Changes in quality of life and pain by treatment

	Pre-treatment	Post-treatment	*P* value
Short-form (SF)-36 score			
Physical functioning	65.0 (46.3–82.5)	65.0 (46.3–76.3)	0.397
Role-physical	56.3 (43.8–76.6)	62.5 (37.5–70.3)	0.103
Bodily pain	42.0 (22.0–72.5)	74.0 (48.5–75.5)	0.012
General health perception	37.0 (31.5–50.0)	32.0 (25.0–48.3)	0.286
Vitality	56.3 (50.0–62.5)	56.3 (43.8–68.8)	0.609
Social functioning	75.0 (46.9–75.0)	75.0 (50.0–90.6)	0.944
Role-emotional	66.7 (50.0–79.2)	66.7 (47.9–83.3)	0.388
Mental health	55.0 (55.0–70.0)	60.0 (45.0–71.3)	0.490
Visual analog scale	4 (3–5)	2 (2–3)	0.001

Data are shown as the median (interquartile range)

**Table 3:** Adverse events and reasons for therapy cessation

	All grades	Grade 3
Hematological, *N* (%)		
Anemia	4 (20.0)	2 (10.0)
Thrombopenia	4 (20.0)	1 (5.0)
Neutropenia	3 (15.0)	0
Non-hematological, *N* (%)		
Fatigue	8 (40.0)	2 (10.0)
Anorexia	5 (25.0)	2 (10.0)
Edema	3 (15.0)	1 (5.0)
Infection	3 (15.0)	1 (5.0)
Hand–foot reaction	3 (15.0)	1 (5.0)
Hypertension	5 (25.0)	0
Alopecia	5 (25.0)	0
Stomatitis	5 (25.0)	0
Fever	4 (20.0)	0
Nausea	3 (15.0)	0
Stomach ache	2 (10.0)	0

	*N*	(%)
Therapy cessation	17	85.0
Reasons for cessation		
Death	7	35.0
Poor physical condition	3	15.0
Disease progression	3	15.0
Adverse events	3	15.0
Patient’s wish	1	5.0

A summary of the adverse events that occurred in at least two patients (10 %) and were classified as grade 3 is shown in Table 2. Hematological events occurred in 11 patients (55.0 %), and two patients required a blood transfusion for severe anemia. There were no patients who were treated with antibiotics and granulocyte colony-stimulating factor (G-CSF) for neutropenia. The most common non-hematological adverse event was fatigue (*n* = 8, 40.0 %). One patient with grade 3 fatigue had to stop this therapy. Anorexia occurred in five patients (25.0 %), and two patients (10.0 %) were classified as grade 3. In regard to sorafenib-related toxicities, although hypertension and stomatitis were common adverse events (five patients, 25 %), they were not severe. A hand–foot reaction occurred in three patients (15.0 %), and it led to cessation of the therapy in two patients (10.0 %); the causes of therapy cessation were severe fatigue and hand–foot reaction in three patients (Table 3). On the other hand, two patients (10.0 %) discontinued treatment after one cycle due to rapid progression of these events; these patients received this treatment as third-line therapy. In addition, one patient stopped the therapy after one cycle at their own request, although they experienced no severe adverse events.

## Discussion

Based on previous in vivo and in vitro studies, various types of molecular-targeting agents have been used for advanced UC in clinical trials [15]. However, there is a general agreement that no single agent or combination regimen led to a decrease in tumor size and improvement in the prognosis in these patients. In addition, several clinical trials of molecular-targeting agents were stopped during the recruitment phase because of unacceptable therapy-related toxicities [16]. In regard to sorafenib, several clinical studies have been performed in patients with UC. For example, single-agent sorafenib was used for the treatment of chemo-naïve UC patients with metastatic disease [17]. However, this study showed that there was no objective response and the median time to progression and survival periods were 1.9 and 5.9 months, respectively. Another report of second-line sorafenib single-agent therapy also showed that it has minimal anti-tumor activity in patients with UC [18]. On the other hand, combination therapy with sorafenib and conventional chemotherapy has been investigated in clinical trials of patients with advanced UC. For example, in 2014, the efficacy and safety of combination therapy of a GC regimen and sorafenib in patients with locally advanced and/or metastasized UC was investigated [13]. This combination therapy was performed as first-line treatment in patients with advanced UC, and their progression-free survival and OS were 6.3 and 11.3 months, respectively, which were similar to those in the CG regimen plus placebo group (6.1 and 10.6 months, respectively). Thus, unfortunately, the efficacy of a single-agent and combination therapy of sorafenib has not been shown in clinical studies. In regard to adverse events, a phase II trial of single-agent sorafenib for 27 patients showed that 18 % of the patients had to stop the therapy because of toxicities [18]. In addition, with a combination regimen of GC and sorafenib, adverse events occurred in 35 of 41 patients (85.4 %) and 25 patients (61.0 %) experienced severe events [13]. In these previous clinical studies, sorafenib was orally administered at 400 mg twice daily [13, 18]. Based on these facts, we decreased the dosage of sorafenib to 400 mg/day in our regimen.

Our study showed that the median (IQR) OS from the start of LD-GPS therapy was 7 (5–11) months. A previous study using second-line therapy with sorafenib for patients with CDDP-resistant UC revealed a median OS of 6.8 months [18]. On the other hand, we previously reported that the median (IQR) survival rate following second-line therapy with LD-GP regimen (GEM: 700 mg/m^2^ and PTX: 70 mg/m^2^ on days 1 and 8, repeated every 28 days) in patients with CDDP-resistant UC was 12 (6–22) months [7]. Thus, the prolongation of survival with LD-GPS regimen was not better than that of other regimens.

Our results showed that the decrease in QoL caused by the LD-GPS therapy is minimal. One of the most interesting findings in this study is the fact that pain was improved by this therapy. This finding was confirmed by two different parameters, including the VAS and bodily pain score of SF-36. In addition, interestingly, the improvement in pain relief not only occurred in second-line therapy, but also in third-line therapy. Based on these results, we suggest that LD-GPS therapy may be useful as both second-line and third-line therapy for the control of QoL, including pain relief.

The frequency and severity of toxicity with this regimen were relatively low. In fact, no patient required granulocyte colony-stimulating factor (G-CSF) and platelet transfusion. Actually, LD-GP therapy resulted in severe leukopenia requiring treatment with G-CSF and thrombocytopenia requiring platelet transfusion in 14.3 and 5.7 % of patients, respectively. In addition, a phase 2 study of sorafenib for advanced UC patients previously treated with one prior chemotherapy showed that grade 4 pulmonary embolism occurred in two of 27 patients (7 %), although our study population had no patients with embolization [18]. This phase 2 study also reported that grade 3 fatigue and hand–foot reaction each occurred in five patients (19 %). While these two non-hematological toxicities also occurred in our study population, grade 3 fatigue and hand–foot reaction only occurred in 10 and 5 % of patients, respectively. On the other hand, many clinical trials of molecular-targeting agents, including sunitinib and bevacizumab, have been reported to have more frequent and severe toxicities [10, 11]. From these facts, when the dosage of sorafenib is increased to more than 800 mg/day, GP plus sorafenib therapy may not be tolerated and safe for advanced UC patients who were previously treated with chemotherapy.

The main limitations of this study are the relatively low number of patients and the uniformity of patients’ clinicopathological features. In this study, we paid special attention to safety and QoL in LD-GPS therapy because no similar regimen has been described in previous reports. In recent years, anti-PD-1 treatment is attracting worldwide attention [19]. However,

On the other hand, we must emphasize the possibility that a decreased dosage of sorafenib may stimulate the malignant behavior in patients with UC. That is, in vitro studies showed that pharmacological concentrations of sorafenib ≥3 μM can inhibit migration and proliferation as well as promote apoptosis; however, low concentrations of sorafenib (0.1 μM) stimulated migration and proliferation of bladder cancer cells [20]. In addition, another recent study also demonstrated that a significant increase in bladder cancer cell viability was detected at a low concentration of sorafenib (2 μM) in vitro [21]. However, there is no general agreement on whether a low dose of sorafenib promotes tumor growth and progression in UC. In fact, in a previous study, a significant increase in migration and proliferation was observed with 0.1 μM of sorafenib, but not with 1 μM [20]. In addition, the stimulatory effect of 0.1 μM sorafenib was found in RT4 and T24 cells, but not in J82 cells. Likewise, in the latter study, an increase in cell viability with 2 μM sorafenib was observed in T24 cells, but not in VMCub1 cells [21]. Thus, more detailed studies are necessary to conclude the relationship between the concentration of sorafenib and the anticancer effects in UC cells. In fact, when our results concerning progression and outcomes are compared with those in other reports using a GP regimen, a negative influence of low-dose sorafenib in patients with UC was not noticed. Similarly, other investigators also reported that the influence of different doses of sorafenib on outcome in patients with UC was not confirmed [13]. However, we are concerned that extremely low concentrations of sorafenib and a variety of factors in the microenvironment surrounding cancer cells may enhance tumor growth and progression in vivo. We support the opinion that the dose of sorafenib must be carefully considered when planning therapeutic regimens including sorafenib in patients with UC.

In conclusion, this study is the first report of a prospective phase II trial to investigate the safety, maintenance of QoL, changes in pain relief, and anticancer effects of LD-GPS regimen in patients with CDDP-resistant UC. Our results indicate that this regimen is safe and well tolerated as second- and third-line therapies. However, the anticancer effects, including the prolongation of survival, were similar or less than those with other previous regimens in a second-line setting. On the other hand, the improvement in pain relief with this therapy in a third-line setting was similar to that in a second-line setting. As such, we support the opinion that LD-GPS therapy is worthy of consideration for patients with CDDP-resistant UC as both second- and third-line regimens.
